# Ethnic Differences, Lung Cancer Risk, and Association of NRF2 Gene Polymorphism with Gemcitabine-Based Chemotherapy

**DOI:** 10.7759/cureus.64849

**Published:** 2024-07-18

**Authors:** Tirumalasetty Devika, Ganesapandian Mahalakshmi, K Mythili, Katiboina Srinivasa Rao, Suresh Kumar Srinivasamurthy, Dubashi Biswajit, Deepak Gopal Shewade

**Affiliations:** 1 Department of Pharmacology, Guntur Medical College, Guntur, IND; 2 Department of Pharmacology, Nandha Medical College and Hospital, Erode, IND; 3 Department of Physiology, Siddhartha Medical College, Vijayawada, IND; 4 Department of Pharmacology, All India Institute of Medical Sciences, Mangalagiri, Mangalagiri, IND; 5 Department of Pharmacology, RAK (Ras Al Khaimah) College of Medical Sciences, RAK Medical and Health Sciences University, Ras AI Khaimah, ARE; 6 Department of Medical Oncology, Jawaharlal Institute of Postgraduate Medical Education & Research, Puducherry, IND; 7 Department of Pharmacology, Jawaharlal Institute of Postgraduate Medical Education & Research, Puducherry, IND

**Keywords:** south indians, rs6721961 c>a, chemotherapy, polymorphisms, gemcitabine, lung cancer, nrf2

## Abstract

Introduction: The cancer burden is rising every year. Lung cancer is one of the most common cancers and non-small cell lung cancer is the most common type. Chemotherapy based on platinum drugs and third-generation nucleoside anti-metabolites such as gemcitabine are used widely. Gemcitabine has a complex metabolic pathway, with many mechanisms contributing to its cytotoxicity. Derangements in the metabolic pathway genes contribute to drug resistance and toxicity with this drug. Association studies including these genetic polymorphisms in the metabolic pathway, clinical outcomes, and cancer risk reported inter-individual differences. Thus, the aim of this study was to ascertain the role of these genetic variants in South Indian cancer patients treated with gemcitabine-based therapy.

Methods: The study was done with 184 healthy volunteers for frequency establishment and 123 cancer patients were treated with gemcitabine-based chemotherapy for response and toxicity assessment. The participants were aged 18-65 years and resided in the southern states of India. DNA extraction was done from the leukocyte fraction of the blood by phenol-chloroform extraction procedures and genotyping was done by reverse transcription-polymerase chain reaction (RT-PCR) techniques to identify DNA repair gene polymorphisms. Tumor response was determined using Response evaluation criteria in solid tumors (RECIST) guidelines and toxicity using Common Terminology Criteria for Adverse Events (CTCAE), version 4.03. The patients were followed up for survival analysis.

Results: The minor allele frequency of the single nucleotide polymorphism (SNP) NRF2-617 C>A (rs6721961) in the healthy population was 12.8%. SNPs were in Hardy-Weinberg equilibrium (p>0.05). Gender-based differences were not observed with the studied SNP in the healthy population and the lung cancer patients. These frequencies of NRF2 were found to be similar when compared to EUR (European) and all the South Asian subpopulations. They are significantly divergent compared to AFR (African), AMR (American), and EAS (East Asian) populations. The minor allele frequency in cancer patients was found to be 14.2% and the lung cancer risk with the SNP studied could not be detected. There was no association found with the response, toxicity, and survival among lung cancer patients.

Conclusion: NRF2, being a multifaced molecule, did not show a significant association with lung cancer risk, response, and toxicity in patients with gemcitabine-based chemotherapy.

## Introduction

NRF2 is a transcriptional protein that activates numerous genes that are involved in cytoprotection [1-3]. In normal cells, this transcription factor is a cap n collar basic leucine zipper molecule. Chemical cancer susceptibility was found in NRF2 knocked-out mice. Interestingly, RNAi-mediated gene silencing of NRF2 expression inhibited abnormal cancerous growth in non-small cell lung cancer (NSCLC) [4,5]. Promoter polymorphisms in the *NRF2* gene have been identified and evaluated for their correlation with carcinogenesis [6]. Poor prognosis was found in squamous cell carcinoma (SCC) patients who were chronic smokers with *NRF2* mutation [7]. This confirms the role of NRF2 in cancer progression. The constitutive expression provides a survival advantage for the cancer cells to invade the distant organs and to undergo changes that lead to chemo-resistance under hypoxia. Increased DNA damage and decreased expression of drug transporters have been observed in cells transfected with NRF2 siRNA [8-10]. Another study showed that cisplatin sensitivity has been restored in human ovarian cancer cells by inhibiting NRF2 function [11].

Assay studies including transient transfection have shown that the polymorphism of NRF2 rs6721961 C>A has significantly altered its protein expression levels and thus the subsequent functions. It has been observed that this SNP is associated with a high risk of causing lung injury. This effect can probably be explained by the fact that rs6721961 C>A is involved in the self-induction of the *NRF2* gene by being present in the antioxidant response element (ARE) of the gene. To explain it further, this SNP initiates the regulation of the positive feedback loop of the activation of the *NRF2* gene and thus modifies its protein expression. As mentioned earlier, by being located at ARE-like loci of *the NRF2* gene, it reduces the binding affinity to target transcription factors. Thus, it is believed that the homozygous variant A/A can attenuate this positive feedback loop of NRF2 and decrease its transcriptional activation [12] It was also found that lung cancer patients who harbor homozygous variant genotype of rs6721961 C>A showed high overall survival compared to the other genotypes [13].

Ethnic differences markedly influence the benefits of cancer chemotherapy [14]. India is home to a diverse population that comes from a range of ethnic backgrounds. Therefore, genetic differences across multiple ethnic groups are expected, and the southern region of India denotes a diverse population [15]. These differences form the main reasons for the varying results and have the potential to skew association study results [16]. Thus, ethnic indicators in addition to common genetic markers are employed as diagnostic and therapeutic tools to fulfill the goal of customized chemotherapy.

In this study, we aimed to establish the normative frequency of NRF2 rs6721961 C>A (-617C>A, 2q31.2 Upstream gene variant). The distribution of the aforementioned variant in the ethnic Asian population has been identified, along with its correlation with lung cancer susceptibility. Furthermore, our objectives included evaluating the commonalities and/or differences among different populations with 1000 genomes and investigating the relationship between NRF2 rs6721961 C>A and response and toxicity in patients with NSCLC.

## Materials and methods

This was a prospective, cohort study conducted at the Department of Pharmacology, Jawaharlal Institute of Postgraduate Medical Education and Research (JIPMER), Puducherry, in collaboration with the Department of Medical Oncology, Regional Cancer Centre (RCC), JIPMER, from June 2014 to June 2017. For survival analysis, the patients were followed up till August 2018 (37 months). The study was approved by the Institutional Ethics Committee (Human Studies), JIPMER, Puducherry, India (approval number: JIP/IEC/2014/4/310). Written informed consent was taken from all the participants.

A total of 307 individuals participated in the study and were divided into the control group which comprised 184 unrelated, healthy individuals aged 18-70 years, who had lived in the southern part of India for three or more generations, and the test group which comprised 123 patients with a confirmed diagnosis of metastatic stage IV lung cancer on gemcitabine-based chemotherapy, and attending the Regional Cancer Centre, JIPMER. The control group included 89 female and 95 male participants, with a mean age of 52.0±10.5 years and the test group included 47 female and 76 male patients with a mean age of 53.5±9.9. Of the 123 lung cancer patients, 47(38.2) were smokers.

Exclusion criteria for healthy volunteers were subjects with impaired liver or kidney function and pregnant and lactating women while the exclusion criteria for cancer patients were: (i) Pregnant and lactating women, (ii) Kidney dysfunction, (iii) Chronic liver diseases (serum creatinine ≥1.5 times normal value, serum transaminase, and serum bilirubin ≥2 times normal value).

Brief history, baseline data including the demographic features, Eastern Cooperative Oncology Group (ECOG) performance status, initial CT, stage of the tumor, biopsy report, and levels of biomarkers if any, and other routine investigations like hemogram and liver and renal function tests were documented. Patients received 1000 mg/m^2^ of gemcitabine on day 1 and day 8 and carboplatin of area under the curve 5 was given in addition on day 1 for four to six cycles. Response Evaluation Criteria in Solid Tumors (RECIST) version 1.1 [17] and Common Terminology Criteria for Adverse Events (CTCAE) version 4.03 [18] guidelines were used to assess response and toxicity in lung cancer patients, respectively. Data from the 1000 Genomes Project from the International Genome Sample Resource (IGSR) (https://www.internationalgenome.org/) was used to compare the allele and genotype frequencies in the studied healthy population with global populations.

Statistical analysis was done using IBM SPSS Statistics for Windows, Version 19.0 (Released 2010; IBM Corp., Armonk, New York, United States) and GraphPad InStat version 3.06 (Dotmatics, Boston, Massachusetts, United States). The two-sided P-value of 0.05 was considered significant. Allele and genotype frequencies were analyzed using the direct gene count method. For the assessment of Hardy-Weinberg equilibrium and frequency comparison between the healthy South Indian volunteers and the 1000 genome populations, the Chi-Square (χ2 ) test was used. Linkage disequilibrium analysis was done using the PLEM algorithm with HaploView software V 4.2 (Informer Technologies, Inc., Los Angeles, California). Association of *NRF2* gene polymorphism and response to gemcitabine-based chemotherapy as well as for case-control analysis, binary logistic regression was used. The association of *NRF2* gene polymorphism and occurrence of toxicities was analyzed using the Chi-Square (χ2) test or Fisher’s exact test. Survival analysis was done by Log-rank and Cox regression test.

## Results

The genotype and allele frequencies in the control group (n=184) were in Hardy-Weinberg equilibrium. The allele frequencies of NRF2 C>A (rs6721961) were 87.2% for C and 12.8% for A. Genotype frequencies were found to be CC 77.7%, CA 19.0%, and AA 3.3%. To establish the normative frequency, the healthy group was compared with the 1000 Genome Project populations which also included healthy participants from different ethnicities. These frequencies of NRF2 were found to be similar when compared to EUR (European) and all the South Asian subpopulations. They are significantly divergent compared to AFR (African), AMR (American), and EAS (East Asian) populations (Table 1). There were no gender-wise differences observed with this SNP (Table 2).

**Table 1:** Comparison of the genotype and allele frequencies of NRF2 rs6721961 C>A polymorphism of the healthy subjects with the 1000 Genome Project populations* N: number of subjects; SI, South Indian, AFR: African; AMR: American; EAS: East Asian; EUR: European; SAS: South Asian, BEB, Bengali in Bangladesh GIH, Gujarat Indian in Houston; ITU; Indian Telugu in the United Kingdom; PJL: Punjabi in Lahore; STU, Srilankan Tamil in the United Kingdom *Data from the 1000 Genomes Project from the International Genome Sample Resource (IGSR) (https://www.internationalgenome.org/)

Present	N	Genotype frequency (%)			Allele frequency (%)		P value
		CC	CA	AA	C	A	
SI	184	77.7	19.0	3.3	87.2	12.8	Ref
AFR	661	88.7	11.2	0.1	94.3	5.7	<0.0001
AMR	347	67.4	27.4	5.2	81.1	18.9	0.011
EAS	504	57.7	35.9	6.3	75.7	24.3	<0.0001
EUR	503	76.3	22.3	1.4	87.5	12.5	0.902
SAS							
BEB	86	66.3	29.1	4.7	80.8	19.2	0.050
GIH	103	71.8	24.3	3.9	84.0	16.0	0.281
ITU	102	74.5	24.5	1.0	86.8	13.2	0.874
PJL	96	79.2	16.7	4.2	87.5	12.5	0.926
STU	102	70.6	27.5	1.9	84.3	15.7	0.333

**Table 2:** Gender-wise genotype and allele frequency distribution of gene polymorphism in the healthy population (N = 184)

NRF2 rs6721961	CC, n (%)	CA, n (%)	AA, n (%)	C, n (%)	A, n (%)	P value
Male (n=95)	72 (75.8)	20 (21.1)	3 (3.2)	164 (86.3)	26 (13.7)	
Female (n=89)	71 (79.8)	15 (16.9)	3 (3.4)	157 (88.2)	21 (11.8)	0.587

The frequencies of alleles in lung cancer patients (n=123) were found to be 85.8% for C and 14.2% for A. The genotype frequencies were CC 76.4%, CA 18.7%, and AA 4.9%. In addition, there were no gender-wise differences observed (Table 3).

**Table 3:** Gender-wise genotype and allele frequency distribution of gene polymorphism in the test (lung cancer) population (N= 123)

NRF2 rs6721961	CC, n (%)	CA, n (%)	AA, n (%)	C, n (%)	A, n (%)	P value
Male (n=78)	61 (78.2)	13 (16.7)	4 (5.1)	135 (86.5)	21 (13.5)	
Female (n=45)	33 (73.3)	10 (22.2)	2 (4.4)	76 (84.4)	14 (15.6)	0.650

Case-control analysis did not find any risk of lung cancer susceptibility with NRF2 C>A (rs6721961) (Table 4).

**Table 4:** Case control analysis of studied polymorphism

NRF2 rs6721961 C>A	Test group (N = 123), n (%)	Control group (N = 184), n (%)	p-value	Odds ratio	95% CI
CC	94 (76.4)	143 (77.7)	1		
CA	23 (18.7)	35 (19.0)	0.9992	0.9997	0.555 to 1.798
AA	6 (4.9)	6 (3.3)	0.4760	1.521	0.476 to 4.860

Of the 123 lung cancer patients in the study, the response was evaluable for 95 patients. The median number of chemotherapy cycles was 4 (range, 1‑6). Among them, we did not find any patients with complete response. Partial response (PR) was observed in 42 patients, stable disease (SD) in 24 patients, and progressive disease (PD) was observed in 29 patients. The objective response rate was 44.2% (PR) and the non-responders were 55.8% (SD and PD). The studied SNPs did not show any significant association with the response to gemcitabine therapy (Table 5).

**Table 5:** Association of NRF2 gene polymorphism with response to gemcitabine-based chemotherapy in lung cancer patients (N=95)

NRF2 rs6721961 C>A	Responders (n=42)	Non-responders (n=53)	Total (n=95)	p-value	Odds ratio	95% CI	
CC	35	35	70				
CA	6	13	19	0.159	0.462	0.158	1.352
AA	1	5	6	0.151	0.200	0.022	1.801

The toxicity profile was evaluated for all 123 patients in the test (lung cancer) group. They were categorized into hematological and nonhematological toxicities. Non-hematological toxicities were mild and manageable. Among hematological toxicities, anemia (62%) was found to be the highest followed by thrombocytopenia (29.2%). Grading of the hematological toxicities was done using CTCAE criteria. Except for anemia, not many patients had grade 3 and 4 toxicities. Few patients underwent blood transfusion and granulocyte‑colony stimulating factor injection administration. There was no significant association observed with the studied SNP (Table 6).

**Table 6:** Association of NRF2 gene polymorphisms with toxicities of gemcitabine-based chemotherapy in South Indian cancer patients (N=123)

NRF2 rs6721961 C>A	Anemia (n=76), n	p-value	Thrombo cytopenia (n=36), n	p-value	Leucopenia (n=20), n	p-value	Neutropenia (n=29), n	p-value
CC	62		27		13		22	
CA	11	0.112	7	0.871	6	0.153	6	0.787
AA	3	0.419	2	1.000	1	1.000	1	1.000

**Figure 1:**
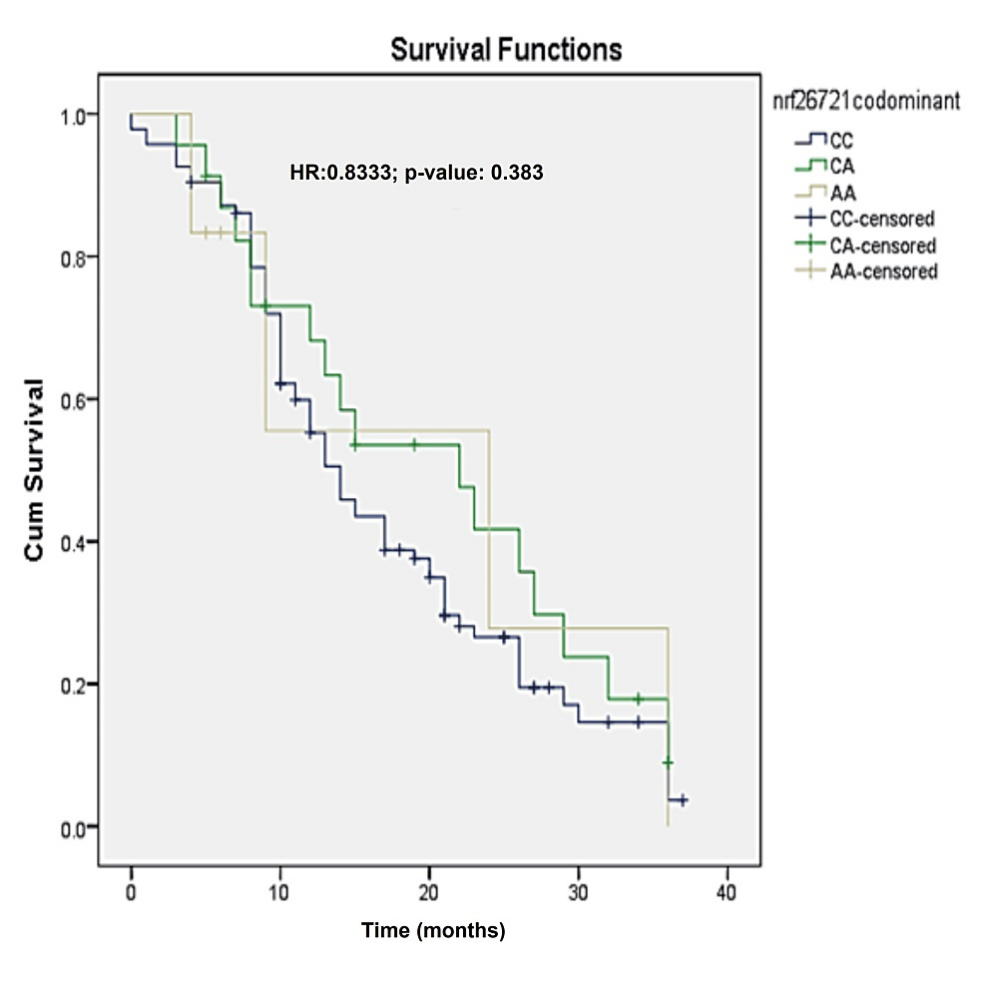
Kaplan-Meier curve of NRF2 rs 6721961 C>A for the lung cancer patients (N=123) HR: hazard ratio; Cum Survival: cumulative survival

Survival analysis was performed, and the median overall survival was found to be 14 months (95%CI 11.0-16.9). Cox regression analysis was performed to find out the association of survival with the SNP. There was no significant association found between the studied SNP and survival in patients with gemcitabine-based chemotherapy. The survival curves for the studied SNP are given in Figure 1.

## Discussion

The study results show that there were significant differences in allele and genotype frequencies between the study population and the 1000 Genome Project populations. Case-control analysis did not show any risk of the studied SNP with lung cancer. Additionally, there was no significant association found between the NRF2 genetic variant with response, toxicity, and survival in lung cancer patients.

Activation of NRF2 is a critical step in the process of cytoprotection as it is involved in the eradication of reactive free radicals. At the genetic level, however, the regulatory pathways involved in activating NRF2 are not fully understood. It is well known that NRF2 activity is regulated by a protein called Keap1 (Kelch-like ECH-associated protein 1). During oxidative stress, this protein releases NRF2, which in turn causes activation of the antioxidant pathway. Thus, any alteration in this regulation can lead to increased oxidative stress that allows cancer cells to acquire different resistance mechanisms for constant proliferation. Thus, NRF2 determines the progression of cancer and resistance to chemotherapy [19].

Genotype frequencies of NRF2 rs6721961 C>A were found to be CC 77.7%, CA 19.0%, and AA 3.3%. These frequencies of NRF2 were found to be similar when compared to EUR and all the South Asian subpopulations. They are significantly divergent as compared to AFR, AMR, and EAS populations. The AFR and Taiwanese [13] population had a lower frequency of 5.7% and 7.1% compared to the present study (12.8%). In the AMR and EAS population, the frequencies are higher (18.9% and 24.3%).

Carcinogenicity may be elicited by either extrinsic or intrinsic activation of inflammatory pathways which results in immunosuppression, thereby creating a favorable background for progression [15]. NRF2 is an important factor that is involved in initiating the antioxidant pathway and protects the normal and also premalignant cells from free radical insults. Because of the continuous accumulation of damaged DNA, there is constant activation of NRF2 during tumorigenesis. Prolonged activation of NRF2 removes the reactive oxygen species along with activation of metabolic pathway genes leading to cellular reprogramming and enhanced proliferation of cancer cells. Constitutive expression of NRF2 also increases the activation of metabolizing enzymes of drugs and thus can confer resistance and poor prognosis in cancer patients [20]. SNPs in NRF2 at the molecular level affect the activation of NRF2. The SNP rs6721961 C>A has been found to be significantly associated with protein expression of NRF2 in vitro and in vivo [12]. This SNP was reported to act through antioxidant response element (ARE), activates NRF2, and thus affects the transcription of transporters and metabolizing enzymes as said earlier [21-23].

The present study could not find any association between genetic polymorphism of NRF2 with response and toxicity to gemcitabine in addition to the risk of developing lung cancer. A Japanese population-based study was done with 387 lung cancer patients to evaluate the influence of NRF2 rs6721961 C>A. Allele frequencies between males and females were found to be statistically significant (22.6% and 28.6%), and, the ratio of homozygous variant genotype in female patients was significantly higher than in males (10.8% and 2.7%, p =0.004). The authors further analyzed by including the smoking status as a confounding variable and found that nonsmokers were carrying a higher frequency of AA genotype (10.4% and 4.5%, respectively, p=0.02). In further analysis, the significance remained only for gender. Interestingly, out of 20 patients with adenocarcinoma, 16 were females and were nonsmokers. In contrast, in males, all were smoking patients [13].

Mutations in epidermal growth factor receptor (EGFR) have been found recently in their association with smoking status. The most common of them are exon 19 or 21 deletions. These derangements were observed frequently in female, nonsmoking lung cancer patients. In addition, they are most commonly observed in East Asians with adenocarcinoma [24,25]. In the current study, EGFR status was evaluated in 30 patients. Among them, 19 patients were positive for either exon19 or 21 deletions at the tyrosine kinase domain. Okano et al. found a correlation between EGFR mutations and NRF2 variant genotype AA in females with nonsmoking status and adenocarcinoma [13]. Another study has found similar results in evaluating the risk of lung cancer where the homozygous variant AA of SNP rs6721961 C>A was significantly associated with EGFR mutations [26]. Both EGFR and NRF2 follow the same pattern of expression in lung cancer patients. Thus, it can be of great value to investigate more and evaluate for any common signaling talk between them.

In the current study, the patients were followed up for 37 months and were analyzed for survival outcomes. The median overall survival was found to be 14 months (95%CI 11.0-16.9). Cox regression analysis was performed to find out the association of survival with SNPs. There was no significant association found between the studied SNP and survival in patients with gemcitabine-based chemotherapy.

NRF2 plays a double role in protecting and destroying the cells/body. Thus, its expression levels and derangements can determine the risk of cancer as well as the clinical outcomes of anticancer drugs by helping cancer cells proliferate and by causing degradation of the drugs. Our study results showed no significant association of NRF2 rs6721961 C>A with survival in lung cancer patients. In an extensive study including 304 lung specimens the immuno-histochemical analysis showed unusually high expression of NRF2, and was correlated with frequent relapse [19]. It has been found that lung cancer patients who harbor homozygous variant genotype rs6721961 C>A showed high overall survival compared to the other genotypes. Lung cancer patients from stages I to IV were included for analysis and it was found that the wild type and heterozygous genotypes were not correlated with survival. Further, stage I alone, adenocarcinoma, squamous, and large cell types were included for analysis which also resulted in a favorable prognosis. The authors reported that all the patients with variant genotype AA and adenocarcinoma had survived over 1000 days after surgical resection without any follow-up treatment [13].

The limitation of this study is that the sample size was small to extrapolate the results. The plasma concentration of drug and its metabolite were not estimated, which could give additional understanding in evaluating the results with the studied polymorphism. NRF2 has various roles and lung cancer is a multifactorial disease with many facets; thus, future studies with multiple biomarkers may be considered in analyzing the better treatment option.

## Conclusions

The present study findings showed remarkable heterogeneity with respect to the genotype frequency of the South Indian population compared to other global populations. We could not get a significant association of the studied polymorphism with lung cancer risk, response, toxicity, and overall survival in patients on gemcitabine-based chemotherapy. *NRF2* has multifarious roles, the polymorphisms of which need to be evaluated on a larger scale to understand the intricate functions in response to gemcitabine-based chemotherapy.
